# Public health determinants of child malaria mortality: a surveillance study within Siaya County, Western Kenya

**DOI:** 10.1186/s12936-023-04502-9

**Published:** 2023-02-23

**Authors:** Thomas Hollowell, Maquins Odhiambo Sewe, Joacim Rocklöv, David Obor, Frank Odhiambo, Clas Ahlm

**Affiliations:** 1 Department of Clinical Microbiology, Infection and Immunology, Umeå University, Umeå, Sweden; 2 Department of Infectious Diseases, Karlstad Central Hospital, Region Värmland, Karlstad, Sweden; 3 KEMRI Centre for Global Health Research, Kisumu, Kenya; 4 Department of Public Health and Clinical Medicine, Section of Sustainable Health, Umeå University, Umeå, Sweden; 5 Heidelberg Institute of Global Health and Interdisciplinary Center for Scientific Computing, University of Heidelberg, Heidelberg, Germany

**Keywords:** Malaria, Public health, Children, Epidemiological monitoring, Demographic surveillance, Child mortality

## Abstract

**Background:**

Malaria deaths among children have been declining worldwide during the last two decades. Despite preventive, epidemiologic and therapy-development work, mortality rate decline has stagnated in western Kenya resulting in persistently high child malaria morbidity and mortality. The aim of this study was to identify public health determinants influencing the high burden of malaria deaths among children in this region.

**Methods:**

A total of 221,929 children, 111,488 females and 110,441 males, under the age of 5 years were enrolled in the Kenya Medical Research Institute/Center for Disease Control Health and Demographic Surveillance System (KEMRI/CDC HDSS) study area in Siaya County during the period 2003–2013. Cause of death was determined by use of verbal autopsy.

Age-specific mortality rates were computed, and cox proportional hazard regression was used to model time to malaria death controlling for the socio-demographic factors. A variety of demographic, social and epidemiologic factors were examined.

**Results:**

In total 8,696 (3.9%) children died during the study period. Malaria was the most prevalent cause of death and constituted 33.2% of all causes of death, followed by acute respiratory infections (26.7%) and HIV/AIDS related deaths (18.6%). There was a marked decrease in overall mortality rate from 2003 to 2013, except for a spike in the rates in 2008. The hazard of death differed between age groups with the youngest having the highest hazard of death HR 6.07 (95% CI 5.10–7.22). Overall, the risk attenuated with age and mortality risks were limited beyond 4 years of age. Longer distance to healthcare HR of 1.44 (95% CI 1.29–1.60), l ow maternal education HR 3.91 (95% CI 1.86–8.22), and low socioeconomic status HR 1.44 (95% CI 1.26–1.64) were all significantly associated with increased hazard of malaria death among children.

**Conclusions:**

While child mortality due to malaria in the study area in Western Kenya, has been decreasing, a final step toward significant risk reduction is yet to be accomplished. This study highlights residual proximal determinants of risk which can further inform preventive actions.

## Background

Approximately 241 million cases of malaria are reported annually, causing an estimated 627,000 deaths in 2020. 95% of all malaria deaths are estimated to occur in the African region [1]. Children are one of the most vulnerable groups for malaria infection and mortality. Of all malaria deaths worldwide in 2020, 77% were children under the age of 5 years [1]. Young children are at an increased risk of both symptomatic infection and developing severe malaria as they have not yet developed full effective partial immunity. Severe malaria can manifest with several symptoms, cerebral malaria (coma) is one of the most fatal. Other symptoms include metabolic acidosis, severe anaemia, acute renal failure, or acute pulmonary oedema. Even when treated, the mortality rate is 10–20% at this stage [2].

Over the past decades, advances have been made in awareness, prevention, and treatment. These achievements are largely due to the UN Millennium Development Goal of reversing the incidence of malaria as well as many global and national programmes dedicated to reversing the incidence and mortality of malaria. Up until 2004 malaria burden was on the rise but since then malaria attributed child deaths have decreased by 31.5% in sub-Saharan Africa [3].

Without an effective malaria vaccine, focus has previously been directed at prevention and treatment of the disease. Prevention projects have mainly focused on public education, vector control, such as insecticide-treated bed nets (ITNs) and long-lasting insecticidal nets (LLINs), indoor residual spraying, and to some extent larval control.

Treatment options have evolved and have become more readily available, and policy has changed to provide the most effective treatment across the globe. This includes treatment of symptomatic malaria as well as intermittent preventive treatment for both pregnant women and to a lesser extent, children under 5 years of age. The availability and usage of rapid diagnostic tests (RDTs) has had a marked increase from less than 200,000 tests distributed in 2005 to more than 400 million distributed in 2020 [1]. *Plasmodium falciparum* drug resistance has been an emerging problem and as a result the preferred treatment has evolved to now recommend artemisinin-based combination therapy (ACT) in most malaria-prone regions, especially in sub-Saharan Africa where all malaria subtypes are present and drug resistance has been an issue [1].

In Kenyan healthcare today, malaria is responsible for 13–15% of outpatient consultations. A total of 70% of Kenya's population live in malaria regions, and 13 million in malaria-endemic areas [4]. The population at risk in Kenya is just over 37 million according to the latest World Health Organization (WHO) World malaria report that estimated over 14 million suspected malaria cases throughout the country in 2020 [1].

Compared to certain regions of sub-Saharan Africa, such as West and Central Africa, Kenya has had a significant reduction in malaria burden nationwide, yet studies show that there are still large differences within Kenya depending on geographical location. The central highland region of Kenya has seen a reduction in malaria cases from 100 cases per 1000 inhabitants in 2003 to nearly none in 2008, while the coastal region and Lake Victoria region still have higher parasite prevalence rates partly due to conducive climate conditions [5, 6].

### Previous surveying of malaria among children in Siaya County, Kenya

The Kenya Medical Research Institute (KEMRI) has been present in Kisumu since 1984, focusing on public health research and infectious diseases. Siaya County is in the middle of the region with the highest malaria endemicity in Kenya and more than 99% of all malaria infections are caused by *P. falciparum* [7–9]. Transmission modelling based on parasitic screening shows the area to have predicted rates of over 40% parasite prevalence year-round [10]. A previous study conducted in the KEMRI/CDC HDSS additionally showed peaks in mortality following periods of rain [11]. In 2008 the overall under 5 child mortality rate per 1000 person-years was 47.1 [12]. One study specifically investigated the under 5 child mortality rates due to malaria in two KEMRI/HDSS surveillance areas (Asembo and Gem) with a total population of 147,988 and showed a decrease in mortality rate from 13.2 per 1000 person years in 2003 to 3.7 per 1000 person years in 2010, indicating that major progress has been made in the region regarding malaria mortality in children under the age of 5 years [13]. In comparison to other causes of death, malaria caused 36.5% of deaths among infants (1–11 months) and 44.1% of deaths among children (1–4 years old) in 2010. Other common causes of death were acute respiratory infections, HIV/AIDS related deaths and diarrhoeal diseases [14]. A previous study investigated slide positivity rates among children aged 0–14 years old who visited health clinics with a history of or documented fever and found the rate to be 52.5% between 2007 and 2012, giving an indication of high malaria incidence in the region [15].

The aim of the present study was to investigate demographic and public health factors and their effect on malaria mortality in children under 5 years of age in the highly endemic area in Western Kenya using surveillance of a large population cohort.

## Methods

### Study area

**Fig. 1:**
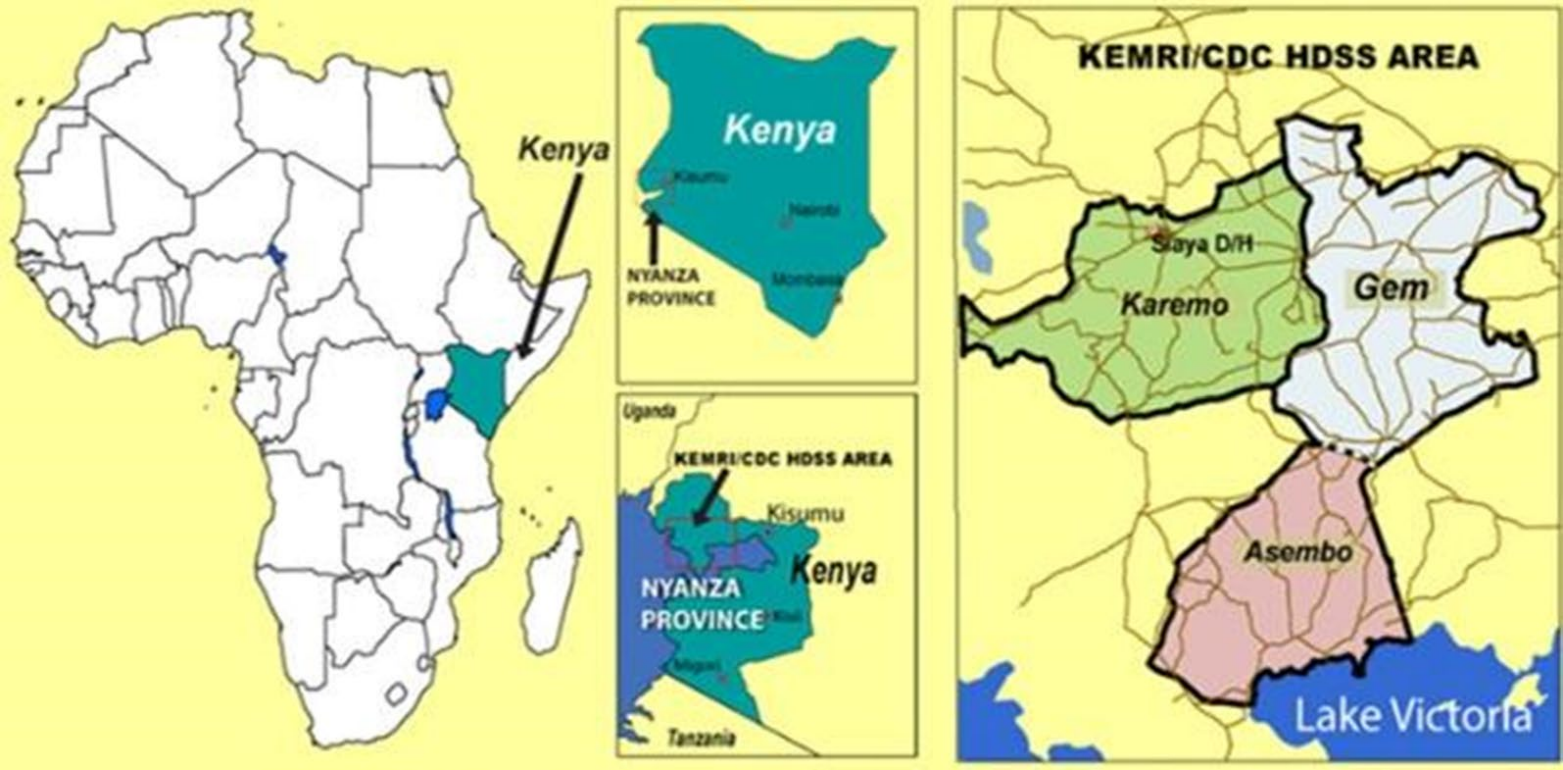
The KEMRI/CDC Health and Demographic Surveillance System (HDSS) study area in Western Kenya (left)

**Fig. 2:**
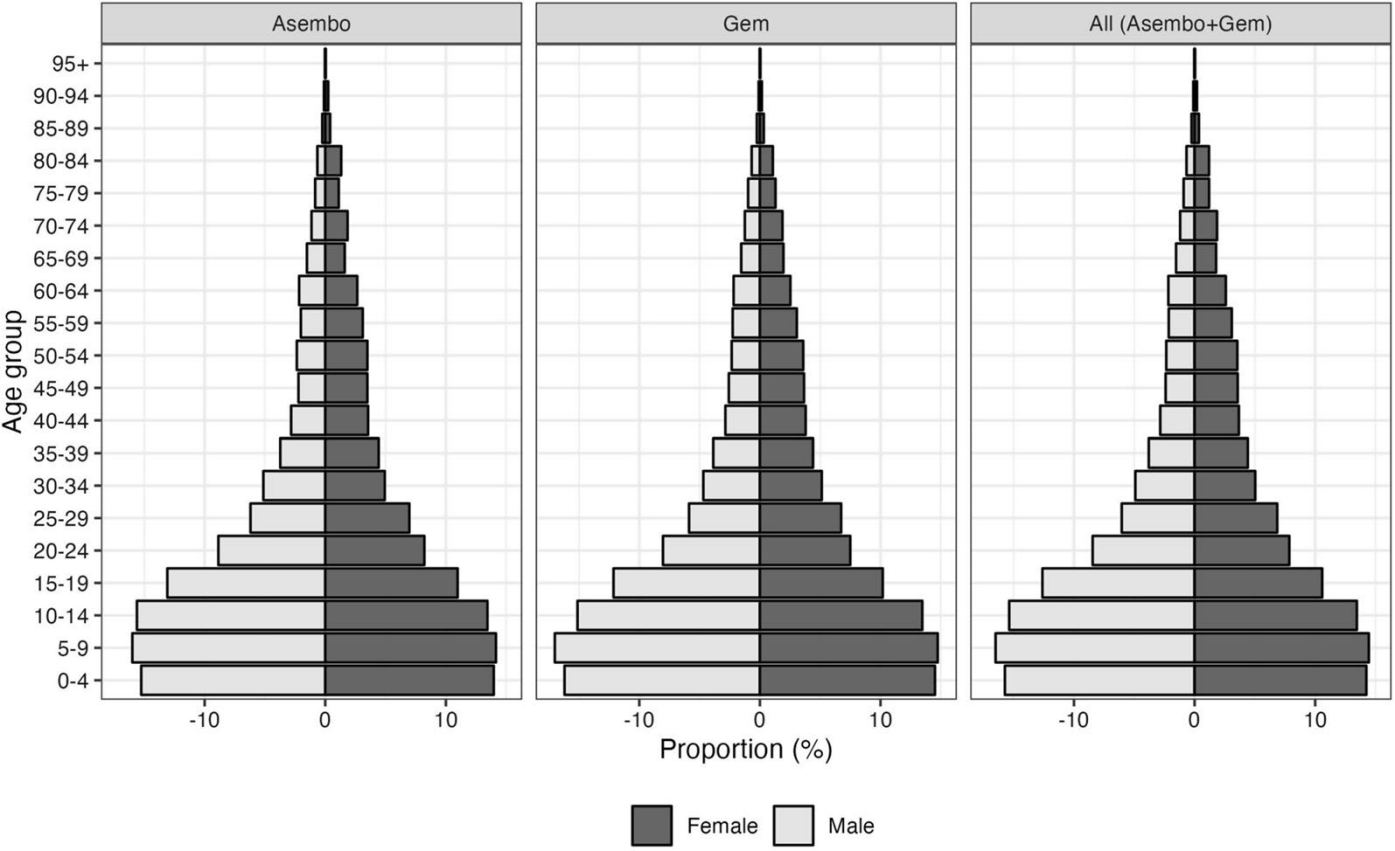
Population pyramid by HDSS areas, Asembo, Gem ad combined (Asembo and Gem) for 2012

The KEMRI/CDC Health and Demographic Surveillance System (HDSS) provides longitudinal population-based demographic information such as burden of disease, accessibility to healthcare facilities, socioeconomic status, household information and family relations, accessibility/usage of public health interventions, and cause of death through verbal autopsy [12]. The HDSS covers three contiguous demographic surveillance areas in Siaya County; Asembo, Gem and Karemo, west of Kisumu, Kenya, on the northeastern shore of Lake Victoria (Fig. 1). It is a rural region with around 45% of the population under 15 years of age and 5% of the population over 65, in 2013 the mid-year population was estimated at 252,570. Figure 2 displays the population pyramid for the year 2012 with a larger base where 15% of the population comprise children below 5 years of age. More than 95% of the population survives on subsistence farming, fishing, and local trading [12].

The surveillance of public health determinants of child malaria mortality was performed in the regions of Asembo, Gem and Karemo (right). During the years included in the analysis, information on migrations, births and deaths were collected from residents three times per year by trained community interviewers, as well as collection of a large variety of demographic factors including household characteristics, ITN usage, education level, and the GPS location of the compounds [12, 14]. Information on educational status and socioeconomic status were collected every 2 years [12]. In addition to the regular surveillance, a system comprising village reporters who report the events as soon as they occur in the community is used to improve timeliness of birth and death registration. After a death notification, the bereaved family is allowed a grieving period of at least 3 weeks, whereby the main caregiver is interviewed to ascertain signs and symptoms of illness prior to death using a standardized questionnaire adjusted to the region [15, 16].

### Study population

All resident children under the age of five excluding neonates between January 1st of 2003 and December 31st of 2013 were included in the analysis. Censoring was done if the child emigrated, was lost to follow-up, turned five before the end of the study period, or still alive by December 31st of 2013. From 2003 until 2007 only Asembo and Gem areas were in the HDSS, Karemo was enumerated in 2007. Thus, for Asembo and Gem, the data runs from 2003 to 2013 while for Karemo, the data runs from 2008 to 2013.

### Verbal autopsy

In most parts of sub-Saharan Africa, Kenya included, there is no reliable system in place to perform and record traditional physician-performed autopsies as there is a lack of adequate number of physicians, proper equipment, facilities, funding, and time. Therefore, a verbal autopsy system is used by the HDSS to derive probable cause of death. The standardized questionnaire, provided by the WHO targeted to children aged 28 days to 14 years asking questions specific to the disease burden of the region was used. The questionnaire is administered to the family of the deceased by community interviewers after a death has occurred in the community. The answers given are then interpreted by InterVa, a probabilistic Bayesian software that provides three probable causes of death ranked by probability given the signs and symptoms. The most probable cause of death was used to extract malaria deaths.

### Data

From the HDSS database the following variables were extracted: individual ID, date of birth, sex, GPS location, compound ID, entry and exit dates, type of event initiating and terminating residency, mother’s ID, mother’s level of education, mother’s date of birth, cause of death, socioeconomic status, household ITN ownership and household size. From household and health care facility geocodes, distance was measured to the nearest health facility, not considering road networks and terrain. The main outcome was malaria death.

A panel dataset with person years of follow up was created by time-splitting each individual exposure time into separate age groups since each individual grows into separate age groups over the study period. The age ranges were defined as 29 days–1 year, 1–2 year, 2–3 year, 3–4 year, and finally 4–5 years, labeled as 29- < 1, 1, 2, 3 and 4, respectively. All individuals under the age of 29 days or older than 5 years were removed from the cohort.

### Statistical analysis

Trends in age specific malaria mortality rates were computed with 95% confidence intervals stratifying by the covariates included in the model. Cox proportional hazards regression was used to model time to malaria death controlling for covariates. All data management and analysis were done using Stata 13.1.

## Results

During the 2003–2013 period a total of 221,929 individual children from 29 days of age up to 5 years of age, 111,488 females and 110,441 males, were followed by the HDSS culminating into 335,801 person years of observation (Table 1).

**Table 1:** Cohort characteristics for study of malaria mortality in children under 5 years of age, Western Kenya

	Person-years of observation	Population (%)	Number of malaria deaths (proportion, %)
Sex			
Female	167,460	111,488 (50.2)	1418 (48.9)
Male	168,341	110,441 (49.8)	1480 (51.1)
Age			
29– < 1	65,574	157,575* (20.5)	1054 (36.4)
1	68,443	157,333* (20.5)	931 (32.1)
2	67,509	152,978* (19.9)	487 (16.8)
3	67,327	150,687* (19.6)	267 (9.2)
4	66,947	149,150* (19.4)	159 (5.5)
Calendar year			
2003	22,033	50,577** (6.6)	245 (8.5)
2004	21,898	50,224** (6.5)	306 (10.5)
2005	21,680	49,484** (6.5)	215 (7.4)
2006	22,055	50,118** (6.5)	171 (5.9)
2007	28,268	70,904** (9.2)	138 (4.6)
2008	37,144	84,993** (11.1)	553 (19.1)
2009	36,578	82,613** (10.8)	395 (13.6)
2010	36,445	82,342** (10.7)	280 (9.7)
2011	36,233	80,617** (10.5)	205 (7.1)
2012	36,619	81,960** (10.7)	182 (6.3)
2013	36,848	83,891** (10.9)	208(7.3)
Distance to health care			
0–1 km	73,111	48,033 (21.6)	466 (16.1)
1–2 km	107,030	70,794 (31.9)	952 (32.9)
2 + km	155,472	102,935 (46.4)	1,479 (51.0)
Unknown	188	167 (0.1)	1 (0.0)
Maternal education			
None	5,104	3,427 (1.6)	56 (1.9)
Primary	227,149	140,879 (63.5)	2,196 (75.8)
Secondary	36,805	24,006 (10.8)	220 (7.6)
Higher	3,459	2,340 (1.1)	8 (0.3)
Unknown	63,284	51,267 (23.1)	418 (14.4)
Mother´s age			
< 20	66,716	50,258 (22.7)	542 (18.7)
20–30	171,807	109,378 (49.3)	1,552 (53.6)
> 30	84,313	47,133 (21.2)	728 (25.1)
Unknown	12,966	15,160 (6.8)	76 (2.6)
Socioeconomic status			
Most poor	54,839	35,785 (16.1)	617 (21.3)
Very poor	60,698	38,694 (17.4)	592 (20.4)
Poor	59,399	38,369 (17.3)	508 (17.5)
Less poor	55,764	36,455 (16.4)	453 (15.6)
Least poor	55,489	36,708 (16.5)	393 (13.6)
Unknown	49,612	35,918 (16.2)	335 (11.6)
Household size			
1–2	104,621	52,445 (23.6)	940 (32.4)
3–4	98,239	70,467 (31.8)	849 (29.3)
5 +	124,727	90,977 (41.0)	1,088 (37.5)
Unknown	8,213	8,020 (3.6)	21 (0.7)
ITN ownership			
None	44,377	30,196 (13.6)	524 (18.1)
1 or more	63,924	39,717 (17.9)	669 (23.1)
Unknown	227,501	152,016 (68.5)	1,705 (58.8)

^*^Total person observations per category during the study period

^*^^*^Total of children aged 29 days–5 years old observed per year during the study period

In total, 8,696 (3.9%) children aged 29 days–5 years old in the population died during the study period. The cause of death was determined to be malaria in 2,898 cases, a total of 1.3% of the study population. Of all causes of death among these children, 33.2% were due to malaria which was the most prevalent cause of death, followed by acute respiratory infections (26.7%) and HIV/AIDS related deaths (18.6%).

The analysis of mortality rates and hazard ratios based on the demographic variables show steadily decreasing incidence rates and hazard ratios. Overall, the incidence and hazard ratio s are considerably higher in the youngest group (29- < 1 year) and declines with aging (Table 2).

**Table 2:** Malaria mortality in children under 5 years of age, Western Kenya

Variable	Hazard ratio (95% CI)	Mortality rate per 1000 person years (95% CI)
Age		
29– < 1	6.07 (5.10–7.22)*	16.1 (15.1–17.1)
1	5.30 (4.45–6.31)*	13.6 (12.8–14.5)
2	2.85 (2.36–3.43)*	7.2 (6.6–7.9)
3	1.60 (1.31–1.97)*	4.0 (3.5–4.5)
4	1 (ref)	2.4 (2.0–2.8)
Calendar year		
2003	1 (ref)	11.1 (9.8–12.6)
2004	1.26 (1.05–1.52)*	14.0 (12.5–15.6)
2005	0.89 (0.73–1.09)	9.9 (8.7–11.3)
2006	0.64 (0.52–0.79)*	7.8 (6.7–9.0)
2007	0.34 (0.27–0.43)*	4.9 (4.1–5.8)
2008	1.04 (0.87–1.23)	14.9 (13.7–16.2)
2009	0.77 (0.64–0.93)*	10.8 (9.8–11.9)
2010	0.54 (0.44–0.66)*	7.7 (6.8–8.6)
2011	0.43 (0.34–0.53)*	5.7 (4.9–6.5)
2012	0.45 (0.36–0.56)*	4.9 (4.3–5.7)
2013	0.46 (0.37–0.57)*	5.6 (4.9–6.5)
Sex		
Female	1 (ref)	8.5 (8.0–8.9)
Male	1.04 (0.96–1.12)	8.8 (8.4–9.3)
Distance to health care		
0–1 km	1 (ref)	6.4 (5.8–7.0)
1–2 km	1.35 (1.21–1.51)*	8.9 (8.3–9.5)
2 + km	1.44 (1.29–1.60)*	9.5 (9.0–10.0)
Maternal education		
None	3.91 (1.86–8.22)*	11.0 (8.4–14.3)
Primary	3.31 (1.65–6.65)*	9.7 (9.3–10.1)
Secondary	2.20 (1.09–4.47)*	6.0 (5.2–6.8)
Higher	1 (ref)	2.3 (1.2–4.6)
Mother´s age		
< 20	1 (ref)	8.1 (7.5–8.8)
20–30	1.11 (1.00–1.22)*	9.0 (8.6–9.5)
> 30	1.05 (0.94–1.18)	8.6 (8.0–9.3)
Socioeconomic status		
Most poor	1.44 (1.26–1.64)*	11.3 (10.4–12.2)
Very poor	1.30 (1.14–1.48)*	9.8 (9.0–10.6)
Poor	1.16 (1.01–1.33)*	8.6 (7.8–9.3)
Less poor	1.15 (1.00–1.32)*	8.1 (7.4–8.9)
Least poor	1 (ref)	7.1 (6.4–7.8)
Household size		
1–2	1 (ref)	9.0 (8.4–9.6)
3–4	1.02 (0.91–1.13)	8.6 (8.1–9.2)
5 +	1.05 (0.94–1.16)	8.7 (8.2–9.3)
ITN ownership		
None	1.11 (0.99–1.25)	11.8 (10.8–12.9)
1 or more	1 (ref)	10.5 (9.7–11.3)

Hazard ratios and mortality rates in relation to health determinants. The reference group for each health determinant is indicated in brackets (ref)

^*^Statistically significant

**Fig. 3:**
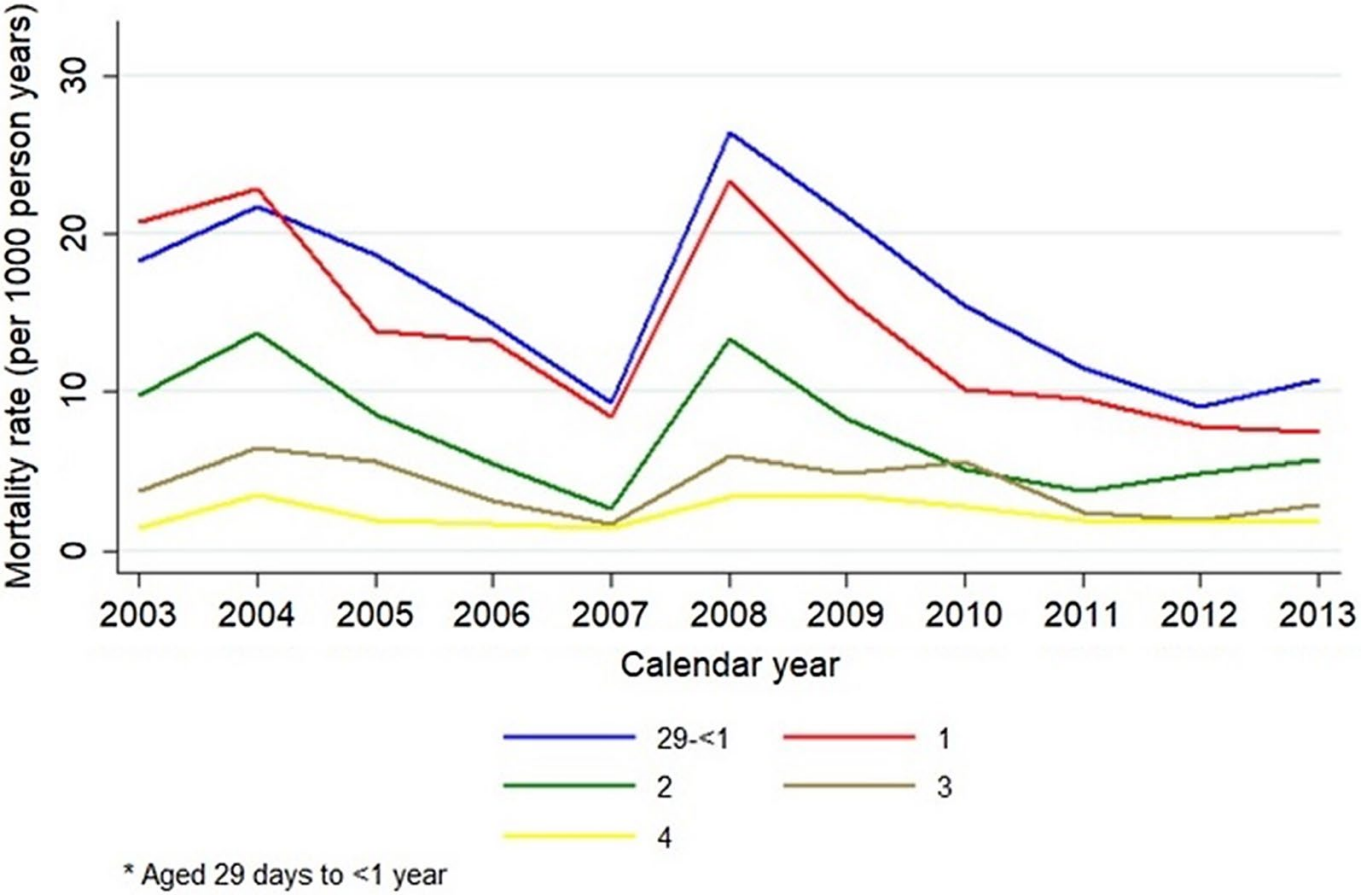
Malaria mortality rates presented by age group per study year for children under the age of 5 years in Western Kenya 2003–2013. The age groups were 29 days–1 year, 1–2 year, 2–3 year, 3–4 year and 4–5 years, respectively

**Fig. 4:**
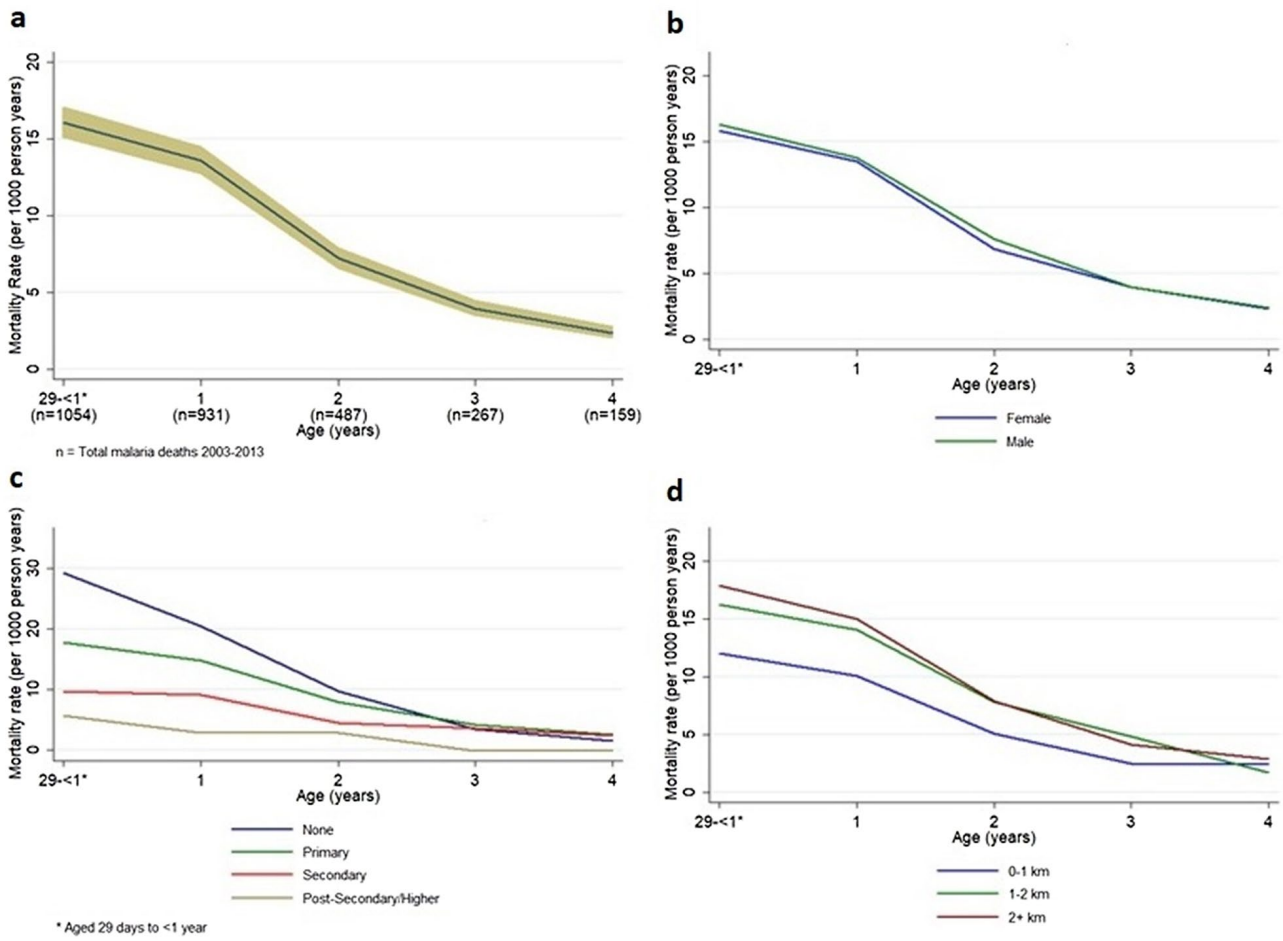
**a**–**d**. Rates of child malaria mortality in a surveillance study, Western Kenya presented by age group for the categories of **a** age, **b** sex, **c** mother’s education and **d** the distance to a health care facility

**Fig. 5:**
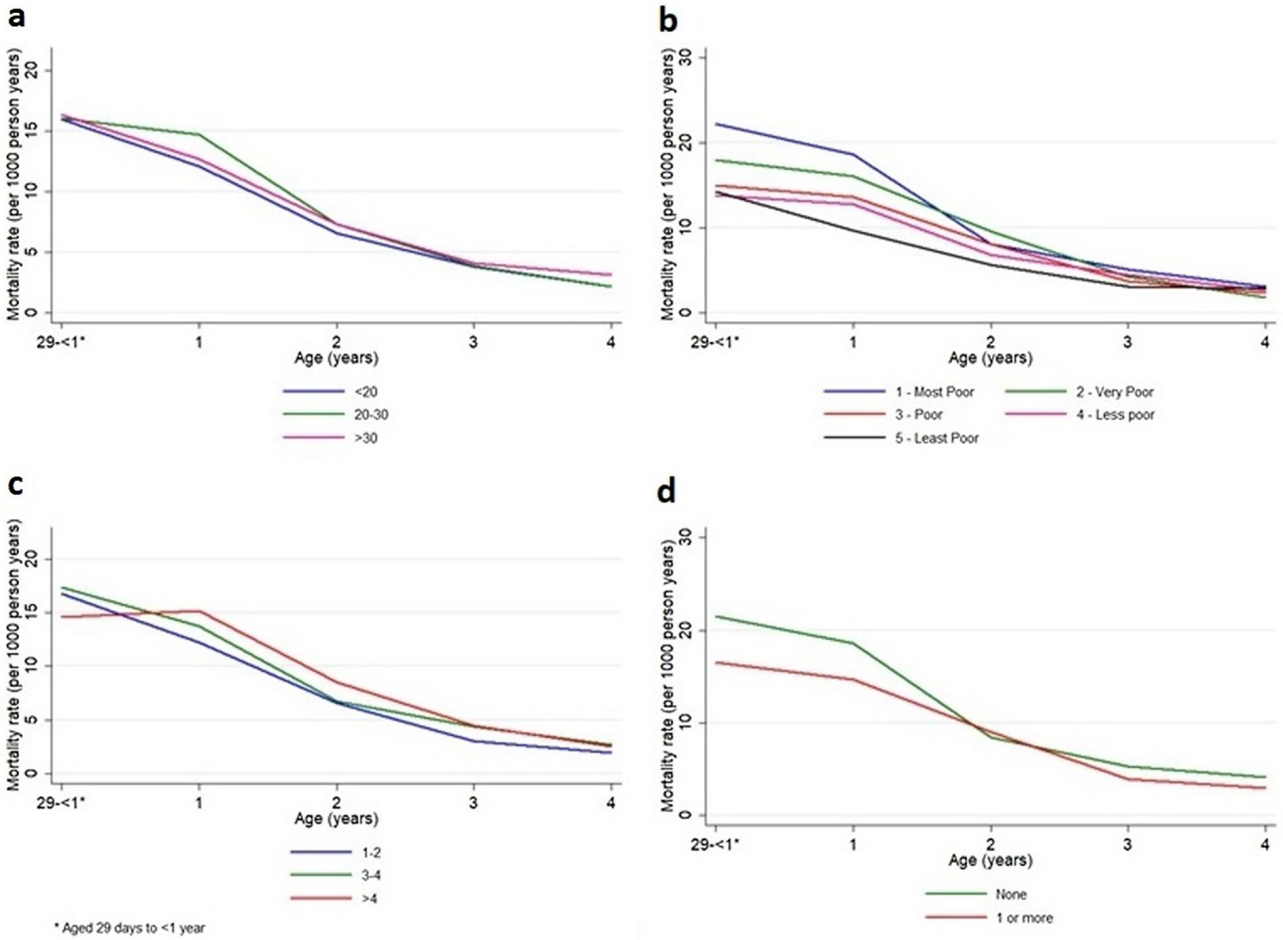
**a**–**d**. Rates of child malaria mortality presented by age group for the categories of **a** mother’s age, **b** socioeconomic status, **c** household size and **d** ITN ownership

Further stratification by calendar year shows steadily decreasing rates and ratios over time, except for year 2008 where a major spike is seen in the incidence (Fig. 3). A lower mortality rate is seen for people with a shorter distance to health care, a higher maternal education and better socioeconomic status (Figs. 4–5). Living closer to a healthcare facility, having a mother with a higher completed level of education, and living in a household with higher socioeconomic status associates to significantly lower mortality rates, as well as gradually decreasing rates and ratios per age category. Smaller deviations from this pattern are seen for males who show a minimally higher rate, as well as for children to younger mothers who tend to have a lower mortality rate (Figs. 4b and 5a). A lower mortality rate is seen in households with at least one child sleeping under ITN (Fig. 5d and Table 1).

## Discussion

Malaria is an important cause of death for children under 5 years of age in sub-Saharan Africa. Although advances in prevention, diagnostic tools and treatment have led to a decrease in deaths, malaria is still responsible for more than one third of avertable deaths in children in endemic areas in Kenya. In this study, the socioeconomic determinants of malaria mortality in the region were investigated. The results highlight important determinants which need to be considered for preventing malaria deaths in children.

The mortality rate in the HDSS area of Siaya County has been steadily decreasing since 2003 yet a sudden increase is seen in 2008. This has been observed in previous studies from the KEMRI/CDC HDSS and can likely be linked to the widespread violence between tribes that followed the 2008 national elections [11, 13, 15, 17]. Many of Kenya's inhabitants were internally displaced and there were frequent stock-outs and disruptions in delivery of ACT, RDTs and other medical supplies within the public healthcare system.

Decrease in healthcare accessibility and implementation of malaria control measures have also been seen elsewhere. A study performed in northern Uganda where the population and local government had endured political instability for more than a decade experienced similar results [18]. Similarly, war and civil unrest in other parts of Africa have led to the resurgence and periodic epidemics of sleeping sickness [19]. In Kenya, post-2008 data shows a further decrease in malaria mortality rates giving further merit to the theory linking post-election violence to the sudden increase in malaria deaths during 2008 and the following years.

Similar increases in malaria incidence and mortality have also been seen after the study period related to the covid-19 pandemic where health care services were disrupted once again. Use of primary health care services decreased after the onset of the covid-19 pandemic by an estimated 28.7% while malaria testing rates are estimated to have decreased by 31.9% due to more frequent RDT stock-outs as well as a decreased willingness to seek health care [20]. As a result, confirmed malaria cases were also decreased during the initial phases of the pandemic [20, 21]. A recent study from Zimbabwe shows increased malaria mortality rates during the covid-19 pandemic [22] as well as WHO estimates of up to 47,000 additional deaths worldwide from malaria in 2020 directly linked to the covid-19 pandemic [1].

The highest mortality rates were found among the youngest children which is consistent with what is known about malaria in children. The first 3 months of life, a child has the benefit of immunity acquired from the mother via birth and breastfeeding. Children aged 3–12 months are more vulnerable and therefore the progression of malaria symptoms can be more rapid and severe than for both younger and older children. According to WHO, a potential issue is also that most anti-malarial drugs lack correct prepackaged dosages for infants which could lead to inaccurate doses being administered, affecting treatment success [2].

There was a slight but non-significant sex difference in malaria mortality, males had a 1.04 (95% CI 0.96–1.12) hazard ratio compared to females. This can be compared to the under-5 mortality rate for all causes in Kenya 2013 where a significant sex imbalance was observed (75 per 1000 person years for males and 66 per 1000 person years for females) [23].

There might be several reasons for the sex differences in mortality, the Kenya Malaria Indicator Survey from 2015 showed that overall, females under 5 years of age are slightly more likely to sleep under a bed net, with 59.1% of females sleeping under any type of bed net, compared to 56.7% for males [24]. A later survey from 2020 showed a more even distribution according to sex, yet even lower rates at 49.0% for males and 50.2%% for females [8]. The survey from 2015 also showed that females under 5 were slightly more likely brought to medical attention (72.2% females, 71.6% males) and to be tested via a finger or heel blood test for malaria when febrile (42.0% females, 36.7% males), and were more likely to receive ACT on the same or the next day (65.1% females, 55.6% males) when febrile [24]. Another important reason for the difference in mortality rates for males and females could be found in the fact that males have a higher parasite prevalence rate, spontaneous rapid diagnostic testing programmes have showed that between the age of 3 months–14 years, 15.3% of males spontaneously tested positive for malaria, compared to 14.0% for females [24]. Thus, the reason for the slightly higher mortality rate among males is likely multifaceted, parasite prevalence rates are slightly higher and healthcare-seeking behaviour tends to favour females slightly as shown in both this study and previous studies.

In the present study, there was a significant lower malaria mortality rate among children whose mothers had a completed secondary school or higher education. The Kenya Malaria Indicator Survey from 2015 concludes that the education level completed by the mother has a large impact on a variety of factors. The higher the general education, the higher the likelihood of sleeping under an ITN, seeking medical attention for a child’s fever as well as the likelihood of receiving the recommended ACT to treat malaria [24].

The United Nations 4th Millennium Development Goal targets the area of education specifically and states that any level of maternal education is beneficial in reducing child mortality, something that Kenya has achieved quite well when considering that merely 1.6% of the mothers included in this study had no completed education whatsoever. The data also shows the benefit of further education with significantly reduced mortality rates among the group with a secondary school or higher education.

Similarly, higher education level is also linked to higher socioeconomic status and lower frequencies of HIV infection compared to those with primary school [25]. It is not entirely clear how large of an impact HIV has on malaria severity as research shows mixed results [26]. Mothers with higher levels of education have also been shown to have higher levels of self-confidence and skill in gathering information as well as increased autonomy within the family in an otherwise male-dominated culture, which can all contribute to increased tendencies to seek medical care at a higher rate and before symptoms become too severe [24, 27].

The results show a clear trend where increasing level of socioeconomic status gradually decreases the child mortality rate. The Kenya National Malaria Strategy published in 2009 showed differences in access to treatment according to socioeconomic status, with 17.3% of children in the lowest wealth quintile and 29.1% in the upper wealth quintile taking an antimalarial drug to treat febrility [7]. However, a follow-up in 2015 showed mixed results for both general anti-malarial drug treatment and specific ACT according to wealth quintile, showing lower rates for those in the highest wealth quintile and peaks in the next-lowest and middle quintiles [24]. Another study showed that 40% of children under 5 with a fever in 4 rural Kenya regions could not afford malaria treatment [28].

The Kenya National Malaria Strategy also includes data regarding use of bed nets nationwide and shows significant differences in usage according to socioeconomic status where 39.2% of children under 5 years of age in lower wealth quintile families sleep under a bed net. Those in the higher wealth quintile bracket are covered at 61.6%. The figures are 29.1% and 44.5% respectively for ITNs and LLINs [7].

In Kenya, national policy states that any child under 5 years of age shall receive free health care, yet many Kenyans are unaware of this policy and therefore do not seek medical attention for their children [29]. Another barrier to seeking healthcare is not being able to afford transport to a healthcare facility [29]. It is common to borrow money from relatives or neighbors, but there is also a reluctance to do so [17]. As previously mentioned, socioeconomic status is also closely linked to education level and HIV infections which may also play a role in the mortality rate differences.

When looking at the mother’s age, mothers under the age of 20 tend to have fewer children dying of malaria compared to mothers aged 20–30 and  > 30, however the differences are small. Several studies show the opposite, that mortality rates among first-born children to mothers 18–21 years of age is high. It is also shown that older mothers have lower child mortality rates [30, 31]. The results generated here are however difficult to generalize due to the very small differences.

The distribution of ITNs and LLINs in a controlled and planned manner is proven to be effective in reducing morbidity and mortality in malaria [1, 5, 32] and the Kenya National Malaria Strategy has set a target goal of at least 80% of the population in endemic areas sleeping under an ITN or LLIN every night [7]. A 2010 study performed during a peak transmission period from the Kisii district (150 km from Siaya) showed that 95% of 670 surveyed households owned at least 1 bed net and owned on average 2.4 bed nets per household. However, only 59.2% of children under 5 years of age slept under a net. Additionally, the study showed that 40% of nets had one or multiple holes [33]. Compared to national data, which states that each household owns on average 0.8 bed nets, large differences appear between regions, however this figure also includes the rather large proportion of Kenyans not living in malaria-endemic areas. More recent data from the Kenya Malaria Indicator Survey finds that 75.3% of children under 5 in the lake endemic region slept under any type of bed net the night before surveying in 2015 [7, 24] and even lower in the follow-up survey of 2020 where only 60.3% of children under 5 slept under a bed net [25].

The results show only a slight benefit of ITN ownership, yet due to the large number of missing data any conclusions are difficult to draw. According to another study, ITN coverage is higher than found in the HDSS data as the study area has been given more attention and has participated in several large studies regarding ITNs over the past decade, thus they have received free distribution of nets both from the government and as part of epidemiological studies at steadily increasing rates since 2003 [34]. The HDSS data records whether children under 5 years of age in the household slept under an ITN the night preceding the visit by the community interviewer, making any conclusions about how regularly the children sleep under an ITN hard to draw.

The distance from the household to a healthcare facility clearly impacts the mortality rates. The results show a statistically significant advantage of living close to a healthcare facility with increasing hazard ratios of living within 1–2 km and 2 + km respectively as compared to 0–1 km distance. Inhabitants of rural Kenya often lack their own methods of transportation and ambulance services are sparse or nonexistent. Additionally, households are often distant from the major road connections. Often, they must walk or rely on motorbike taxis or friends and relatives for transportation in emergency situations. A study performed in four Kenyan counties looked at the proportion of inhabitants who travelled to a health clinic run by the government and recorded the distance travelled. The study shows that the closer a household lives to a clinic, the more likely they are to travel there for care and that 56% of the patients will travel to their nearest healthcare facility [35].

An interview-based study performed in 3 African countries, including Western Kenya, identified distance to healthcare facilities as a major issue in seeking treatment. Participants said that they most often had to travel by foot, carrying their sick child, as other methods of transport were too expensive. This often leads to delays in seeking treatment and the child worsening further during transport [17]. Another study performed in the KEMRI/CDC HDSS investigating distance of residence and clinic attendance found similarly that increasing distance to health care facilities acts negatively on the rate of clinic visits [35].

The Kenya Malaria Communication Strategy outlines the goals for improvement in educating the Kenyan population regarding malaria risks and symptoms as well as improving healthcare-seeking behaviour and providing information and assistance regarding preventive measures. It identifies the treatment-seeking behaviour as an obstacle, as many patients self-treat at home before seeking care at a healthcare facility, delaying treatment and in some cases receiving incorrect treatment when visiting a traditional healer or dispensary rather than the recommended government or faith-based clinics [36]. The average time before reaching health care was over 2 days after onset of symptoms in a study from 2009 which also shows deficits in public perception regarding correct treatment and treatment-seeking behaviour [28]. The Kenya malaria indicator survey from 2015 found that in the endemic lake region, 65% of febrile children under the age of 5 years sought medical attention, with 59% leaving a blood test, 55% receiving anti-malarial treatment and 52% treated with ACT [24].

The Kenya National Malaria Strategy summarizes that general knowledge about malaria is at a 95% level in Kenya, however only 10% of the population know about the increased risk of anemia and its associated symptoms and complications and the dangers of malaria infection to infants and pregnant women [7].

There were some limitations in the present study. The ITN data was available from 2007, therefore all data from 2003 to 2007 are listed as unknown when it comes to ITN status, there is a total of 68.5% of individuals listed with an unknown ITN status during the entire study period and there is additional data post-2007 with unknown ITN status. There was missing data in the categories of household size, socioeconomic status, maternal education, and maternal age. Data collection for sex, GPS location and age were however complete.

Even though the proportion of missing data was relatively high for some variables, there were significantly lower mortality rates related to maternal age, socioeconomic status, and household size.

There is a potential risk of recall bias as the verbal autopsy system has certain flaws compared to the golden standard physician-performed autopsies. A large study from the Nairobi, Kenya HDSS showed that the verbal autopsy results only corresponded to the physician-reported cause of death in 31.6% of cases involving children under 5, however the main discrepancies stem from indeterminate cases of tuberculosis, diarrhoeal disease and HIV/AIDS related deaths, the results are much more accurate compared to physician-performed autopsies for among others malaria, meningitis, and pneumonia. The study concludes that the InterVA-4 system for analysing verbal autopsy results is a method effective for analysis of cause of death on a large public health scale, especially considering that there is no other available and effective measurement of cause of death on a community level [16, 21, 37, 38]. Another study from Uganda concluded that verbal autopsy methods for determining malaria deaths have an acceptable diagnostic accuracy when used at a population level, especially in areas with high malaria transmission, and was actually found to underestimate the proportion of deaths due to malaria at high transmission sites. The study found a sensitivity rate of over 50% and a specificity of over 88% for malaria deaths [39]. The KEMRI/HDSS study area has a similarly high malaria prevalence and it is possible that verbal autopsy results could underestimate the actual amount of malaria deaths among the children in this study area as well.

Progress has been made mainly through increased ITN coverage, intermittent preventive treatment in pregnancy, diagnostics and improved pharmaceutical treatments for manifest malaria. These steps taken to reduce malaria mortality are proving effective and have begun to reverse the incidence and mortality rate of malaria, however reaching the entire population of malaria-endemic areas with these measures is problematic. Many still do not have knowledge regarding symptoms and recommended preventive interventions as well access to correct diagnostic possibilities and treatment regimens. The HDSS study area houses several healthcare clinics due to the large amount of research that has been conducted here over the past decades and the region has enjoyed increased international attention and aid. Many other rural Kenyan regions do not have the possibilities nor the infrastructure for similar healthcare development and could very well be worse off than the population studied in the KEMRI/CDC HDSS.

## Conclusions

The large KEMRI/CDC HDSS population base and long-term data collection allows large-scale analysis of the socioeconomic determinants of malaria incidence and survival in children under the age of 5 years. The results presented show that the burden of malaria has reduced in the region, yet it remains a significant cause of death, especially among infants. Shorter distance to healthcare, higher maternal education and higher socioeconomic status were all determinants of significantly improved survival.

Future preventive efforts should consider these factors to achieve further reductions of malaria mortality in young children.

## Data Availability

The data used for the analysis can be requested through the KEMRI/CDC HDSS steering committee.

## Abbreviations

ACT: Artemisinin-based combination therapy
CDC: Center for disease control
HDSS: Health and demographic surveillance system
ITNs: Insecticide-treated bed nets
KEMRI: Kenya Medical Research Institute
LLINs: Long-lasting insecticidal nets
RDTs: Rapid diagnostic tests
